# Following the (e-)Medication List From Pre-Admission to After Discharge at Norwegian Hospitals: An Actor–Network Analysis

**DOI:** 10.1177/10497323251340934

**Published:** 2025-05-19

**Authors:** Henriette Lauvhaug Nybakke

**Affiliations:** 1 Norwegian Centre for E-health Research, 60519 University Hospital of North Norway, Tromsø, Norway; 2 Department of Social Sciences, 8016 University of Tromsø – The Arctic University of North Norway, Tromsø, Norway

**Keywords:** medication list, actor–network theory, material semiotics, rapid ethnography, transitions, primary healthcare, hospitals

## Abstract

This study explores the medication list as a complex and multiple object in transitions of care between primary healthcare and hospitals in Norway. Medication lists exist in various forms—digital and physical—across institutions and systems, serving diverse purposes yet often failing to align. These discrepancies contribute to medication errors, particularly during hospital admission and discharge, recognized as high-risk points in patient care. Utilizing a rapid ethnographic approach, the research involved 130 hours of participatory observations and semistructured interviews with 32 healthcare professionals across various settings. Guided by a sociological lens and informed by actor–network theory, this article tells a story about interactions, practices, and relations surrounding medication lists. The findings highlight the challenges of achieving a single, definitive medication list and advocate for a coordinated model that acknowledges the complexities and variabilities inherent in medication management. This research underscores the importance of understanding the dynamic nature of medication lists and their role as both intermediaries and mediators in Norwegian healthcare.

## Introduction

This study examines the trajectory of patients’ medication lists from pre-admission through hospitalization and post-discharge at Norwegian hospitals. In 2017, the World Health Organization (WHO) launched a five-year global initiative called *Patient Safety Challenge: Medication Without Harm*. Medication errors pose a significant financial burden on healthcare systems worldwide and are among the leading causes of patient harm (WHO, 2023). One of the target areas is medication safety in transitions of care, as unintended discrepancies affect almost every patient transitioning between care settings (WHO, 2019). These discrepancies are particularly common among elderly patients and those with polypharmacy, largely because of communication gaps during care transitions (Johnson et al., 2015). A study in Sweden found that elderly patients, on average, experience two medication errors in each transfer between primary and secondary care (Midlöv et al., 2005). These errors can lead to preventable readmissions, medical harm, and, in some cases, even death (Hesselink et al., 2012; Uitvlugt et al., 2021). Medication use and medication history are essential components of patient assessment during hospital admission (Cornish et al., 2005).

Most patients experience changes to their medication list during hospitalization, but some of these may be undocumented or partially documented, leading to medication discrepancies (Graabæk et al., 2019). General practitioners (GPs) often regard the medication list as the most valuable part of hospital discharge summaries, yet these summaries frequently contain deficient or poor-quality medication information (Caleres, Strandberg, et al., 2018; Karapinar et al., 2010). Several studies have highlighted gaps in the transfer of medication information during transitions between primary and secondary care. For instance, Foulon et al. (2018) found that information transfer often lacks important medication information. A study on elderly patients transitioning from hospital to primary care revealed that discharge reports are often delayed or lost (Caleres, Bondesson, et al., 2018). In a quantitative study of discharge summaries in Sweden, medication discrepancies are most commonly observed in the infectious medicine, oncology, and surgery departments (Caleres et al., 2020).

The complexity of the medication list is particularly evident in the hospital setting, where patient medication information is spread across six to seven different information technology systems in seven Norwegian hospitals (Norwegian Board of Health Supervision, 2021). The Norwegian Board of Health Supervision (2016, 2024) identified medication management as a critical vulnerability. To address these challenges, the Norwegian government aims to implement a shared medication list nationwide. However, the findings of this study suggest that, while new technology may address some issues, it is unlikely to be a panacea and could introduce unforeseen challenges.

This study explores transitions of care and information sharing between primary care and hospitals and within hospitals but focuses mainly on a very important object in Norwegian healthcare—the medication list. Broadly defined, a medication list encompasses any complete record of a patient’s medication—whether digital, physical, or mental. While previous research on medication lists has primarily taken a biomedical approach, this study adopts a sociological lens and leans on actor–network theory (ANT) sensibilities, exploring the interactions and complexities surrounding medication lists in transitions of care. This study aims to investigate the trajectory of medication lists—from pre-admission through hospitalization and post-discharge.

## Methods

**Figure 1.**
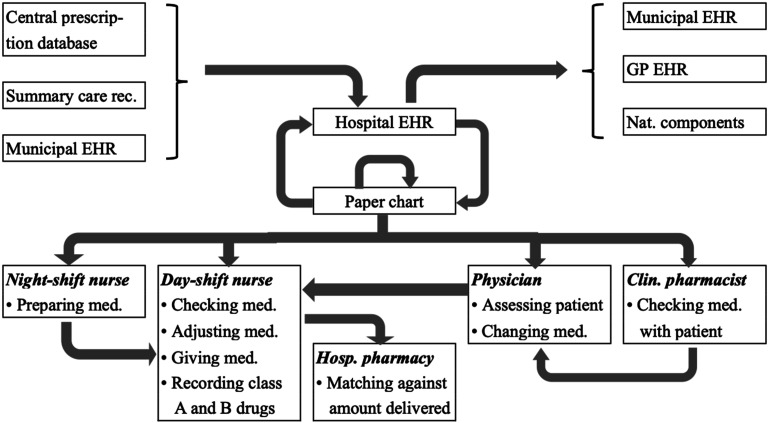
The process of medication management at a Norwegian hospital (Hertzum & Ellingsen, 2023).

This study is part of a larger project that explores medication management practices within the Norwegian healthcare setting. In Norway, municipalities manage primary care, while regional authorities manage specialized hospital services. GPs are typically self-employed but operate under contracts with municipalities (Steihaug et al., 2017). The process of medication management at a Norwegian hospital is simplified by Hertzum and Ellingsen (2023) and presented in Figure 1.

Employing an exploratory qualitative design, this study draws on ANT principles, with inspiration from material semiotics, to examine the relational dynamics between human and nonhuman actors—in this case, medication lists and health professionals. Rapid ethnographic fieldwork was used as the primary method (Vindrola-Padros & Vindrola-Padros, 2018), comprising 130 hours of observations in hospitals, nursing homes, and home care and 29 semistructured interviews with physicians, clinical pharmacists, nurses, and healthcare assistants.

### Actor–Network Theory

Actor–network theory was my companion throughout the fieldwork, or perhaps, I should call it *after-ANT* or material semiotics, aligned with the approaches that influenced my analysis and writing, particularly those of Mol (2002) and Law and Singleton (2005, 2014). The project started with foundational readings on ANT by Latour (2005), which shaped my initial fieldwork approach. ANT challenges traditional distinctions between human and nonhuman actors, conceptualizing objects, such as medication lists, not as passive representations but as actants that shape and are shaped by networks (Latour, 2005). This approach focuses on how entities gain agency through relations. Following the ANT argument that knowledge is not about uncovering a singular truth but about tracing associations, this study explores how medication lists are continuously made and remade through interactions across primary and hospital care. While ANT does not adhere to a fixed epistemology, it takes an empirical, descriptive stance, emphasizing *following the actors* rather than imposing predefined categories. Instead of seeking an objective truth about medication lists, this study focuses on how they are used, interpreted, and transformed through healthcare settings. Rather than reducing complexity, ANT embraces it, providing tools to map how networks stabilize and destabilize.

By following the medication list across institutions, I examine its role as both an intermediary and a mediator (Latour, 2005). At times, it functions as an intermediary, transmitting information unchanged. Other times, it acts as a mediator, actively transforming healthcare practices by introducing discrepancies, requiring reconciliation, or influencing decision making. Using thick descriptions, this study highlights how healthcare professionals navigate these shifting realities. Drawing on Mol’s (2002) concept of ontological multiplicity, I challenge the assumption that the medication list is a single object viewed from different perspectives. Rather, it exists as multiple coexisting realities that emerge through practice.

### Data Collection

The fieldwork was conducted in primary care at two Norwegian municipalities and two hospitals in two health regions between February 2023 and November 2023. One municipality was located in northern Norway with approximately 22,000 inhabitants, while the other was located in southern Norway with approximately 150,000 inhabitants. The project received approval from municipal health and welfare leaders, who selected the institutions to participate. Each institution designated a contact person who was responsible for scheduling observations and identifying potential interviewees. These contact persons were informed to recruit a diverse range of healthcare professionals to capture a comprehensive view of practices, preferably healthcare professionals who had experience with or were involved in medication management.

Participatory observations were conducted over a total of approximately 130 hours: 35 hours in two home care bases, 39 hours in three nursing homes, and 56 hours at one hospital. At the hospital, observations were made at the medical ward, at the surgical and orthopedic wards, and in the emergency room (ER), both day and night. The observations occurred in these settings based on prior information about where medication management commonly occurred and which institutions were most central. Additionally, access was granted by these institutions’ leaders. The observations were documented in real time through handwritten notes, with reflections added post-observation. These notes were subsequently transcribed into Word (Microsoft Corporation, 2023a). Informal interviews (e.g., casual conversations) were handled in the same way. During observations at one of the hospitals and one of the home care services, I was given the same scrubs (uniform) as the nurses, which helped me blend in, although the participants always consented to my presence. The observations were both active and passive, depending on the setting (e.g., active in the break rooms and passive during clinical work).

For the fieldwork, the situations that were possible to observe were limited because of patient confidentiality and ethical considerations. Health professionals are skilled in observing their patients’ and their own practices, although their stories, like most others, can be contradictory and biased. Interviews were conducted with different healthcare professionals across the observed institutions, in addition to one hospital and several GP offices where only interviews were conducted. A semistructured interview guide was created for each profession and workplace, with different versions further developed as new information emerged to inform the questions. At nursing homes, three doctors, six nurses, three healthcare workers, and one social worker were interviewed. In the home care services, interviews were conducted with three nurses and one healthcare worker. Additionally, three GPs, one medical secretary, and one nurse from the GP offices were interviewed. Interviews were also conducted at two hospitals with one junior doctor, five nurses, and four clinical pharmacists. In total, 29 semistructured interviews with 32 health professionals were conducted, with an average length of 47 minutes.

While most interviews took place in person, some were conducted remotely via Teams (Microsoft Corporation, 2023b) or over the phone. All interviews were audio-recorded and transcribed verbatim. The original language of the interviews and fieldnotes was Norwegian, and the translation of quotes and observational notes was conducted after the analysis.

### Ethics

Ethical approval was obtained from each hospital’s data protection officer (University Hospital of Stavanger, ID: 3791-3791, and University Hospital of North Norway, ID: 02852) and the Norwegian Agency for Shared Services in Education and Research (ID: 313244). The project was evaluated by the Regional Committees for Medical and Health Research Ethics and was deemed exempt from further ethical review. Approval to conduct the study was obtained from all participating institutions. All employees were informed about the project, data anonymity and storage, and the researchers’ contact information through email or other communication channels. All participants were verbally provided with information about the study. Written informed consent was obtained from all interview participants, while verbal consent was obtained from the participants during observations. No patient data were collected, and during observations, patient confidentiality was ensured.

### Analysis

The analysis was inspired by ANT, particularly the processes outlined by Tryggestad et al. (2013), Justesen (2017), and Demant and Ravn (2017). My approach was inductive, beginning with a thorough familiarization with the data and evolving into a comprehensive analysis. Using NVivo 12, I traced the medication list across observed practices to map its network and interactions. I then constructed a timeline integrating observation notes and interview excerpts. This timeline is neither linear nor seamless but reflects the medication list’s evolving role through a sequence of events and interactions.

## Findings

The data analysis can be read as a story—one in which each action and situation leads to the next. However, unlike a traditional narrative, this is a fragmented story capturing the fluid and unpredictable nature of the medication list’s journey across contexts. The structure aligns with ANT’s methodological commitment to tracing how actors emerge and transform within a network. It consists of a prologue, which details the period before hospital admission, followed by three acts, which cover key hospitalization events, and concludes with an epilogue, which focuses on the post-discharge phase.

### Prologue: Before Hospital Admission

General practitioners are legally responsible for keeping patients’ medication lists updated and providing accurate lists to other health professionals as needed. If a GP admits a patient to the hospital, they are expected to send an updated medication list. However, this task can be disruptive, especially during emergency admissions outside regular hours, as one GP explained:

If a GP admits a patient, the medication list must already be in order. Usually, when it’s an emergency admission, it comes outside the work schedule. You always have little time, so everything you do is delayed for the rest of the day or until lunch. (GP 2)

When a patient is admitted without consulting their GP, the responsibility often shifts to the patient to provide the hospital with details about their medications. The medication list becomes a key actor, the absence of which introduces uncertainty and increases the risk for errors. For those taking a few medications or who have a clear understanding of their prescriptions, this is typically unproblematic. However, when patients cannot articulate their medication regimen or lack accurate knowledge of their prescriptions, the inaccessible list creates instability.

If a patient is admitted, like acutely admitted, without having been to see me, then, as I understand it, it’s up to the patient themself to inform the hospital about what they’re using, and there’s, of course, a high risk of mistakes. (GP 3)

While patients can bring a GP-provided medication list on paper to the ER, those receiving home care often lack easy access to this information, according to the GPs. Observations of home care services revealed that, although patients may have paper lists at home provided by home care, these are not always promptly sent to the hospital by home care staff or not brought by ambulance personnel. If notified that a patient is admitted, home care typically sends an electronic admission report, which includes the medication list if they are managing the patient’s medications.

When we’re notified about an admitted patient, we send an admission report to the hospital. Included in that report is the medication list we have for the patient. (Nurse 5, home care)

However, the material and temporal properties of digital systems introduce friction, especially during evenings or weekends when no nurses are in the office. During emergencies, hospitals can call home care for immediate information, but the ability of the medication list to *move* across contexts depends on multiple intermediaries. On a Monday morning, I sat with a nurse in the home care office as she was doing administrative work. The nurse turned to the computer to show me what an admission report looked like. The screen displayed an e-message from the hospital about a patient who was admitted, and the hospital requested that the home care service send an admission report with the medication list. The message, however, had been sitting there for three days, confirming how digital messages, although instantaneous in theory, are shaped by work routines and staffing structures.

Patients can also be admitted from nursing homes. Here, the medication list undergoes another transformation, shifting between formats and intermediaries. Nursing home doctors make efforts to ensure that medication information reaches the hospital by sending lists both electronically and in paper form. One doctor explained this as follows:

I write a referral that I send electronically, and the medication list is included. I always print it out and send it with the patient in paper form so that they receive it in two forms. Just to be sure. (Doctor 1, nursing home)

However, technical infrastructures introduce asymmetries in information flow. Some nursing homes lack the ability to send e-messages to hospitals directly, although they can receive messages from them. One doctor described how this inconsistency forces them to rely on paper lists, which hospitals often discard because of electronic health record (EHR) policies. At times, medication lists from nursing homes are sent with ambulance personnel. Nursing home staff need to know exactly where to send digital lists, and mistakes can occur.

Sometimes, it [the medication list] can be sent digitally, but sometimes, they [the nurses] send it on paper. They print the medication list and send it along with the patient (…). We have to assess whether emergency help or medical services are needed … But it’s often sent incorrectly. [laughter] So, perhaps it’s best to … that we can protect ourselves against such errors by sending it when the ambulance comes to pick up the patient. (Doctor 2, nursing home)

If the hospital lacks a current medication list, doctors must act as translators, reconstructing the medication lists from fragmented sources. Alternatively, if the patient has previously been admitted to the hospital, the medication list stored in the EHR system becomes an authoritative yet potentially misleading actant, as it may be outdated and likely incorrect.

### Act 1: The Emergency Room (ER)

For patients with planned hospitalizations, their medication lists are usually organized before they are admitted. However, for those admitted through the ER, a junior doctor must create a new medication list based on existing sources for their hospital admission. During my observation of a junior doctor in the ER, he explained his work to me and voiced his frustrations. At one point, he described his *perfect world*—a world where one accurate and universally accessible medication list exists, eliminating the need to cross-check multiple sources. With a sigh, he expressed his doubt about this possibility and that even if it came true, he was unsure if he would trust the list. This skepticism reveals how digital infrastructures, despite their promises, remain contested.

The junior doctor used three main sources for medication information: (1) the summary care record, (2) the prescription intermediary (an electronic prescription system between practitioners and pharmacies), and (3) the patient’s own account. When I asked which source was the most reliable, he replied sternly that none was completely trustworthy. “You should always use the holy trinity,” he insisted, meaning a combination of all three sources. Another junior doctor confirmed this approach in a later interview.

We’re not the patient’s GP, and the GP, who might’ve known the patient for 20 years, writes to the patient, “use as before.” The patient might understand it, the GP understands it, but for an admitting doctor with a patient who has suddenly deteriorated and can no longer communicate, it is … very difficult … Okay, you can assume, based on typical dosages …, but you can’t be 100% certain about it. And that’s where the gray areas start to appear. (Junior doctor 1, hospital)

In a conversation with a clinical pharmacist, she mentioned the *golden standard*—the practice of checking all available sources, as trusting only three sources was inadequate. She explained that sometimes, there was simply no single correct medication list because discrepancies between systems, patient memory, and healthcare routines continuously reshape it. At one hospital, clinical pharmacists can perform medication reconciliation in the ER, but a doctor must officially approve the final list. I interpreted the clinical pharmacist’s observations as highlighting how some patients act as key mediators by providing reliable information, while others contribute to uncertainty because of a lack of awareness or cognitive impairments. In these cases, pharmacists must piece together their medication histories from various systems. The clinical pharmacist described situations in which patients receive a double medication dosage because of a lack of information in the system:

But, eh … if it happens … patients come into the ER who, … have delirium or something, [and] it’s impossible to talk to them, they’re the ones who manage their own medications, there are no sources other than the summary care record, and they can’t complete it [medication list] in the ER, then we just have to do our best to enter what we think they’re taking. (…). (Clinical pharmacist 1)

Another challenge arises with medications administered at outpatient clinics, which, according to another clinical pharmacist, are recorded only in the EHR system of that specific outpatient clinic at the hospital. For example, new biological drugs given at regular intervals are noted only in outpatient records. As a result, the pharmacist must rely on patients’ recollections or admission notes that might contain hints about their medication schedules. Another point of frustration involves patients receiving home care nursing. Ideally, home care nurses should send an updated medication list with the patient to the ER, but this rarely happens. Clinical pharmacists must call home care nurses to obtain the list. One clinical pharmacist hoped for a standard practice.

Because I think that as soon as they arrive in the ER, it’s important to know what medications they’re using. (…) Eh … so there, eh … yes, I don’t quite understand … why they should wait until… until they … get a notification that they’re admitted before sending the list. It should ideally be sent automatically, preferably as a written, physical list with the patient if they’re admitted. If they know they’re being admitted, they should have a medication list with them. (Clinical pharmacist 2)

Similarly, a junior doctor highlighted the difficulty of reconciling medication lists because of the misalignment between GPs’ working hours and ER admissions and the inaccuracy of existing records:

The GP is at work between 08:00 and 16:00. When does the patient come in? At all hours of the day and night. So, most of the time, yes, you can’t completely reconcile the medication list with 100% certainty … That’s the challenge. (Junior doctor 1, hospital)

When they do receive the medication list from the GP, however, it is rarely updated. If the GP has admitted a patient, they send the medication list, but clinical pharmacists and junior doctors often perceive it as incorrect, so the hospital does not use the list. A clinical pharmacist elaborated on this issue:

… but you know that those lists … referrals and such that come from the GPs, you can almost never fully trust them because it looks like the GPs—when they write the medication lists—include almost everything they’ve prescribed over the last few years … without necessarily updating it for changes or removing medications that were previously used but are no longer in use, which are still on the list from the GP … but there may be some things there that are important to clarify with the GP. (Clinical pharmacist 2)

Once the medication list is considered complete and approved by a doctor, it is printed out and sent with the patient to the ward. When a medication list is approved by a doctor, it is ready to be used by the nurses for administering medications to the patient. At this point, the medication list is temporarily stabilized and made authoritative within the hospital setting. However, further discrepancies may emerge when the patient’s condition changes or when additional information becomes available.

### Act 2: The Hospital Ward

Medication lists must be approved before patients enter the hospital ward. However, there are exceptions when the medication lists are sent unapproved or incomplete, often while awaiting information from home care services or nursing homes. A clinical pharmacist emphasized that the priority is ensuring that the most important medications were in order. The pharmacist explained that doctors sometimes copied the medication list from the last admission when lacking information, which can lead to the patient receiving incorrect medications based on outdated information. The practice of copying turns the medication list into a mediator, which shapes treatment decisions not through active human deliberation but through its own material persistence in the system. Although such instances are reported when discovered, the clinical pharmacist expressed empathy for the doctors, who must review patient history, medical conditions, and diagnoses while trying to find accurate medication information. Consequently, they may choose the path of least resistance in completing the medication list quickly.

The clinical pharmacist also noted that while there had been some improvements in how doctors annotated what needed follow-up on the ward, many cases still involved copying and pasting from earlier records.

Things are just copied from previous lists, and it’s not clear when those were taken, whether it’s from five days ago or six months or a year ago, and the source of that information isn’t indicated either. (Clinical pharmacist 2)

This issue is compounded by the fact that medication reconciliation often occurs after the patient has already been admitted to the ward, although it should ideally be completed in the ER. The delay embeds an element of temporal dislocation, as past prescriptions are projected into the present, sometimes overriding newer medication decisions. This delay can lead to the use of inaccurate medication lists, potentially affecting patient safety. One clinical pharmacist detailed their experience with this as follows:

I’ve corrected quite a few medication lists, especially when we’re on the ward. Not in the ER but on the ward, we often reconcile the day after they’ve been admitted. And there’s already a medication list in place. There’s a lot to sort out. (Clinical pharmacist 1)

During my observations on the ward, I examined medication lists, trying to understand what was written and what the symbols meant. Lists are written on an A4 piece of paper, on both the front and back. At the top is information about the patient, followed by the medications listed point by point. Some medications are enclosed in brackets, while others are marked in pink and yellow. Some are on a sticker stamped on top, and there is correction fluid in several places. Additionally, as space runs out, Post-it notes are used. A paper medication list is supposed to last six days, after which it should be updated in the EHR system, and a new paper list is printed. However, this is often neglected, leading doctors to write in the margins or on Post-it notes. These notes on the margins and handwritten additions function as unregulated intermediaries, extending the life of an outdated document while simultaneously increasing its instability. Each of these material elements—markers, stickers, and correction fluid—serves as nonhuman actants, participating in ongoing negotiations of medication accuracy. A clinical pharmacist pointed out that medication lists are underdocumented in the EHR system, as they are not scanned before completion. Doctors usually write the active ingredients, but nurses help one another by writing the brand names. A nurse remarked that the doctors are not good at writing clearly on the list and explained that a pink marker indicates changes, brackets mean that it is only for that day, and yellow means that it has been discontinued. This was confirmed by a junior doctor:

I’ve always found it difficult to interpret these charts [medication lists]. And then, you know, there are different codes. So, you’re supposed to use a pink marker when there’s a medication change, and you should use a yellow marker when something is discontinued, but sometimes, it’s difficult to visualize because some people forget to use a marker, and then they don’t think there’s a registered change, but there might be a change after all. (Junior doctor 1)

When asked about medication lists, one nurse recounted an incident in which she asked the doctors whether a patient should continue a medicine. The doctor replied that if it said “continue,” it was meant to be continued. However, the patient was not supposed to continue that medication, which was noted in the EHR system but not on the paper medication list. Here, the paper list and the EHR system emerge as competing actants, each exerting its own influence on the flow of clinical decisions. The nurse also mentioned that handwriting can be difficult to interpret and that many pharmaceuticals have similar names. The format of the medication list also varies by department and hospital. For example, when they receive copies from other hospitals, the quality is often poor, with little information about what the patient has been taking or when. One nurse mentioned that there were historical reasons for the current format of medication lists but did not elaborate, instead highlighting ongoing issues with inadequate medication history and as-needed medications, as well as insufficient space to write dosages and times.

During a patient’s hospital stay, the clinical pharmacist reviews some of the patient’s medication lists and often feels like a detective navigating digital journal notes. Although accessing the paper medication list is easier, only one version exists, which everyone is trying to obtain. The digital system might indicate that a patient is using zero medications, while the paper list shows 14. This contradiction signals a breakdown in translation between paper and digital actants, forcing health professionals to reconcile divergent sources of authority. The clinical pharmacist, who rotates between different wards every other week, faces difficulties in reviewing all patients’ medication lists because of personnel shortages. As a result, the selection of lists for review is somewhat random. Additionally, clinical pharmacists can only suggest changes rather than make decisions, and they must rely on doctors to implement any necessary updates. Therefore, despite restrictions, pharmacists often make copies of the paper medication list to review it more thoroughly before providing feedback to the doctors.

No, in DIPS [the EHR system], there are just all the notes that doctors, nurses, pharmacists, or other healthcare personnel have written. So, you have to scroll back in time to the date it was written. It’s sorted by date. You can search for a specific department, but if a PLO [e-message] from home care comes in, for example, it gets added to the date they sent it. So, there isn’t really an updated list in DIPS in a way. (Clinical pharmacist 1)

The medication list is also discussed during pre-rounds, which vary in terms of participants. The senior doctor has the authority to approve changes. During my observations, two junior doctors, one senior doctor, and two nurses were present in one pre-round. The doctors sat in front of stationary computers with the EHR system, quickly navigating through pages while I struggled to keep up with what they saw or read. As they started discussing the medication lists, one junior doctor was handed messy-looking lists and was tasked with fixing them, while the others continued. The medication lists themselves have become resistant actants, disrupting workflow and demanding human intervention. A nurse reflected on this process:

I feel like maybe the doctors aren’t very good at going through patients’ medication lists, while we’re the ones who, in a way, hand out the medications and have a very clear overview of what medications the patients are on. So, if we give a message, it might take a little while before anything gets done. We might have to repeat the message more often so that they’ll … go through the medication list. (…). (Nurse 7, hospital)

### Act 3: Discharge From the Hospital

During my fieldwork, everyone I spoke to mentioned that junior doctors primarily drafted discharge reports, including the most recent medication lists, before a senior doctor approved the reports for sending to the appropriate institutions. However, sometimes, nurses send e-messages containing only the medication list from an unapproved version of the discharge report, as they cannot update or approve the medication list themselves. In these cases, the medication list itself serves as an intermediary, circulating independently of human oversight and carrying traces of its previous states.

While observing in the nurses’ break room, I noted that at least three patients were being discharged either to nursing homes or their own homes with scheduled home care services. One nurse sat in front of the computer and checked their patients’ digital medication lists in the EHR system. She commented that it was sometimes easier to just send the patient on to the nursing home and have the discharge report follow later. In this case, the patient and the medication list become decoupled actants, each following separate trajectories in the discharge network. As several nurses gathered, they began discussing whether to send the report as an e-message or print it to send with the patient. Reviewing the medication list, they noted that it was still incorrect, despite previous efforts to get the doctors to update it accurately. The medication list, resistant to correction, shapes decisions through its incomplete or erroneous state. Finally, one nurse decided to send it as is, reasoning that the doctors would need to correct it later. They remarked that reviewing and managing the medication list should be the doctor’s responsibility, not the nurses’, adding with a sigh that it sometimes felt like they were *babysitting* everyone.

In some cases, clinical pharmacists also reviewed the medication lists, but they did not systematically go through every discharge report for quality control. They only did so for certain patients with whom they were directly involved, meaning that for most patients, medication list accuracy still depended on the doctors.

Nurse reports are concise summaries focused on patients’ immediate needs after discharge to ensure that they receive appropriate care. The nurses explained that when the discharge report was ready, it was sent along with the patient. When asked if they had ever copied the medication list from an unapproved discharge report into the nurses’ report, they admitted that it happened occasionally. One nurse noted that they might only copy the medication list from an unapproved discharge report if they were confident that it was finalized.

We write a nurse report, uh … where we … while we have information about the patient, we also have part of that report where we include the medications at discharge that, uh, the doctor … I mean, we essentially paste that part of the discharge from the doctor, so when it’s approved, we include it in the nurse’s report. So that, uh … home care services and nursing homes receive an updated medication list. (Nurse 6, hospital)

Yet, the discharge process itself remains somewhat of a *black box*, involving a network of actors potentially taken for granted and therefore not fully explored. The discharge process is an assemblage of human and nonhuman actants—doctors, nurses, computers, printed reports, and digital records—engaged in a process of translation in which information is shaped, transferred, and occasionally distorted. To follow the medication list throughout discharge, I would have needed access to other areas, such as meeting rooms, doctors’ offices, and their daily rounds. At one point, I found myself standing outside the junior doctors’ office, trying to catch a glimpse through the glass. Inside, they sat in a row of stationary computers, and above them was a sign with a large, yellow smiley face that read, “If you complete your discharge report on time, there will be cake.”

### Epilogue: After Discharge

When a patient is discharged back into municipal care, discharge reports, especially medication lists for home care nurses, GPs, and nursing home doctors, often become sources of confusion. These discharge reports should include the patient’s new and updated medication lists following hospital treatment. However, they are not always sent promptly upon discharge, or sometimes, they never arrive at all. This is a significant problem for institutions tasked with managing patients’ medications, such as nursing homes and home care. The medication list in the discharge report, as an actant in the healthcare network, mediates the transfer of critical medication information. However, its delayed arrival disrupts the translation of medical decisions across different healthcare settings, forcing nurses and doctors to compensate for its failure.

So, we can risk that … Or we can assume that if a patient is discharged, they’re often told by the hospital to be here two days later, but there’s no paper, no information, nothing prepared … uh … (…). (GP 2)

During an observation with a nurse at a home care service, she explained that they are expected to rely on the hospital’s list, as it is the most recent. However, the hospital’s response is at times so slow that the discharge report from a previous stay arrives only during the patient’s current stay. The e-message system, designed as an intermediary to bridge this communication gap, introduces another layer of fragmentation. It operates in parallel with discharge summaries, leading to conflicts in medication management and forcing home care nurses into the role of mediators who actively negotiate between conflicting lists. They sometimes receive a medication list through e-messages, and then the discharge report arrives two to three weeks later. By then, the medication list in the discharge report may not match the medication list received through e-message. I observed two nurses working on a discharge summary, with one updating the digital system and the other reviewing the list from the hospital. They went over each medication, comparing it to the medication list they had on record, and found significant changes that the hospital had not marked as new. Some changes even seemed illogical, so they had to call the hospital or send e-messages to the GPs to clarify.

Often, we experience that when patients are discharged, the medication list in the discharge summary says “as before” … Let’s say, for example, that the patient had Paracetamol 500 mg three times a day on our medication list. Then, on the medication list that comes from the hospital, it says, “As before. Paracetamol 1 gram three times a day.” And I wonder why … And this can apply to several medications. It says “as before,” and the dosage is listed, but it doesn’t match what was on the patient’s previous medication list, which we sent to them. (Nurse 5, home care)

By reintroducing outdated dosages, the medication list forces nurses into additional work, transforming them into mediators who must manually correct the system’s translation failures. Another nurse mentioned that the hospital often neglects to specify dosage or quantity, which then requires follow-up. This process is time consuming and delays patient care, as contacting the hospital can be a lengthy process.

It’s very easy for the hospital to forget that it needs to specify the dosage and amount and … So, we end up having to call it back, and it takes up a lot of time. (…) The worst part is when it doesn’t provide the correct medical details. It can take a very long time before we can get in touch with a doctor and figure out what they want us to do here. (Nurse 4, nursing home)

When home care nurses seek clarification from GPs, they encounter another problem—GPs have usually not received the discharge reports yet. One GP mentioned that typically they would receive the report two to four weeks after discharge, but sometimes, it could take up to three months. This delay leaves them without the necessary information when nurses inquire about a patient’s medication. Thus, the medication list from the hospital, expected to function as a stable intermediary, fails to enact its agency in a timely manner, forcing GPs and nurses to reconstruct medication histories through alternative networks of communication.

Very often, there needs to be tidying up after a hospitalization or when the patient has been to another doctor. It often ends up that we must do the tidying up that another doctor should have done, for example, at the hospital. The home nurse often sends us an e-message and asks, “What should they actually be on now?” They often receive a temporary discharge summary in which the new medications are listed, but we haven’t received it. (GP 2)

The GP further explained that they are often mistakenly expected to have an up-to-date medication list, despite lacking the latest information from the hospital.

Often, people see it as the GP’s task to have an overview of the medication list for a patient. Also, if doctors at other institutions have added something or removed something, we’re supposed to ensure that it’s correct. This is a completely wrong notion. We really don’t have the opportunity to do that. (GP 3)

The challenge is further compounded by the existence of multiple medication lists. GPs must navigate these inconsistencies carefully to avoid errors, especially when significant time has passed since the last update.

The home nursing service has its list, I have a slightly different list, and it’s not exactly the same as the hospital’s list. So, I have to be extremely alert so that there are no mistakes if, for example, six months have passed. And then, I’ve forgotten that I’ve read it. I have to assume that I’ve forgotten it. (GP 1)

At nursing homes, the situation is equally challenging, particularly on weekends, when fewer staff are available to catch potential errors in the medication list. As one nursing home doctor explained, discharge summaries are recurring sources of frustration:

Perhaps the biggest frustration is the discharge summaries from the hospital, where it’s very obvious that something is wrong. (…) In the worst case, patients arrive on a Friday evening, and the whole weekend passes before someone realizes that something is wrong and takes action. (Doctor 1, nursing home)

While certain departments at the hospital have improved the clarity of their discharge summaries, these summaries sometimes use outdated medication lists rather than the nursing homes’ latest versions. This problem arises even after a recent requirement for doctors to indicate changes in discharge summaries, often leading to incorrect information when the hospital bases its updates on old records.

When we discharge a patient to their homes, they follow that list. But if the patient then returns to the hospital, the new list doesn’t follow them. The hospital uses the list from the first stay. All the changes made in the meantime don’t follow the patient. We’ve seen this several times (…): “This medication didn’t agree with the patient and shouldn’t be given.” And yet the patient was back on it again. (Doctor 3, nursing home)

This inconsistency can also result in patients receiving incorrect medication dosages during hospitalization. A hospital nurse recalled a recent case in which a patient from a nursing home was admitted and later discharged. During the patients’ stay, the nurse discovered that the hospital’s medication list did not align with the list from the nursing home.

When she was admitted, the doctor must have relied on the medication list from an earlier discharge summary … We’ve experienced several times that in the summary care record, there can be many different medications listed … She ended up receiving the wrong doses of some medications throughout her stay. (Nurse 10, hospital)

## Discussion

Before a patient is admitted to the hospital, their medication list exists in multiple forms, spread across institutions, systems, and practices in primary healthcare. These lists are not passive documents but active participants in shaping medical practice, influencing decisions and structuring care interactions. Additionally, the patient’s role in correcting these lists is often minimized, despite their lived experience of medication use. Upon the patient’s admission, junior doctors or clinical pharmacists attempt to consolidate these disparate lists into one, although they may not have access to all versions. Transitions of care, while risky, can serve as *checkpoint*s for ensuring accuracy. However, the result is often a fragmented list, with errors persisting into the hospital stay.

Within the hospital, at least two distinct medication lists typically coexist. Changes made on paper during ward rounds may not be reflected in the EHRs, the prescription intermediary, or the summary care record, leaving health providers in primary healthcare reliant on discharge summaries. These lists not only represent medication use but are also enacted in different ways, depending on their formats, their users, and the infrastructures sustaining them. The agency of the medication list itself becomes apparent when discrepancies arise, forcing clinicians to reconcile inconsistencies and make judgment calls on which source to trust. As presented in this study, patients rarely have a single, unified medication list. Instead, multiple lists exist across institutions and formats, each serving a distinct purpose, yet this multiplicity poses challenges. Thus, the medication list is not a singular, stable entity but an assemblage enacted differently across settings.

### Actors Not Followed

Actor–network theory suggests that researchers follow the actors to trace the networks that sustain objects, such as medication lists. However, when the list exists simultaneously in digital records, printouts, and even mental schemas, ANT’s limitations become evident. Law and Singleton (2005) described their research on alcoholic liver disease as chasing a moving target that also shape-shifts. Similarly, the medication list is an actor that defies simple categorization, taking on different forms and functions depending on its context. It is enacted differently by pharmacists, doctors, and nurses, each interacting with and modifying it in ways that shape its reality. In this sense, the medication list is not only multiple but also contested, as its meaning and reliability are continually negotiated by those who use it.

Junior doctors have been identified as central actors in the work with medication lists, particularly through their role during admission and discharge. However, their absence from direct observation creates a black box. Despite considerable effort, only one junior doctor was interviewed in the current study. This matters because the critical work of reconciling and synchronizing medication lists remains largely unexplored. This invisibility highlights a broader issue in healthcare research—crucial processes often occur behind the scenes, shaping outcomes in ways that are not immediately visible. These are processes that WHO (2019) emphasized as important during care transitions, yet they remain somewhat of a black box for researchers, politicians, and health professionals not included in the transition of care.

Law (2006) suggested that absence is not merely a limitation but a feature of research on messy, complex objects. Researchers cannot fully encompass everything. Instead, they must accept and critically reflect on the exclusions inherent in their methods. Rather than striving for completeness, they should focus on the partial, fragmented nature of research, embracing the messiness of multiplicity. In this view, absence is not only a methodological challenge but also an active force that shapes what can and cannot be known about medication lists.

### One List to Rule Them All

If research methods are messy, what does this imply for the politics of creating a single, unified medication list to solve these problems? The Norwegian government is gradually implementing its shared medication list project, which is currently being piloted in selected municipalities. This centralized digital system is intended to eliminate the fragmentation and inaccuracies that arise from having multiple lists scattered among different institutions. However, this effort to create a singular truth overlooks how objects, such as medication lists, are not merely documented but are also enacted in practice. Will standardization truly address fragmentation, or will it introduce new forms of rigidity and inefficiency?

Consolidating all the *mess* into a single digital system does not necessarily resolve the challenges of multiple medication lists. One critical factor often overlooked is the temporal lag between policy and practice—the time and effort required to align workflows with a unified system’s demands. Moreover, the lived realities of healthcare work suggest that standardization often fails to account for informal, adaptive practices that are crucial for patient safety. While the shared medication list project holds promise, its success hinges on addressing practical realities. For example, if healthcare professionals already struggle to send discharge summaries on time, digitizing the process alone will not necessarily accelerate it. Another significant risk is that informal workarounds and manual processes are in place (Graabæk et al., 2019). These informal practices are actors themselves in the network, influencing how medication lists are enacted and resisting full incorporation into a standardized system. If these practices are not considered, the shared medication list could create a *false sense of security* by giving the appearance of comprehensive accuracy. This tension between political ambitions for efficiency, strict mandates, and resource limitations raises a broad question: Are we striving for an ideal that is inherently unattainable? Efforts to improve safety and standardization must be rooted in practical realism rather than idealism. As Law (2004) stated, we should not try to eliminate messiness but coordinate it, recognizing that the medication list is not a static entity but a dynamic and constantly shifting network of interactions.

### Limitations

This study has several limitations. First, while ANT provided a useful lens for analyzing the medication list as an active participant in healthcare practices, it did not account for cognitive processes, such as how professionals mentally navigate multiple lists. Theories, such as distributed cognition, and cognitive artifacts could offer additional insights into the use of medication lists as cognitive tools. Second, medication errors were not directly observed, limiting the analysis of how discrepancies translate into patient harm. While professionals reflected on errors, their frequencies, causes, and consequences remain uncertain. Third, patient interactions were not observed because of confidentiality constraints, thereby leaving a gap in understanding their role in medication reconciliation. Future research should explore cognitive perspectives and direct error observations and ethically include patient perspectives for a comprehensive analysis.

## Conclusion

This study is one of many stories about a very important object in Norwegian healthcare—the medication list. This work highlights the multiplicity of the medication list as it moves across transitions of care between primary healthcare, hospitals, and within hospitals. Rather than existing as a singular, stable object, it is distributed across institutions, digital systems, and physical formats, with each version reflecting the diverse priorities of the healthcare providers who use it. While this multiplicity enables flexibility and adaptation to context-specific needs, it also introduces risks of fragmentation, inaccuracies, and discrepancies, which particularly come to light during admission and discharge. Politically, the ambition to implement a shared medication list reflects a commitment to resolving challenges by striving for a single, unified source of truth. However, this effort may risk oversimplifying the realities of healthcare, in which multiple lists serve distinct purposes. The pursuit of a single, definitive list is, in many ways, a false ideal because healthcare is inherently complex, and standardization alone cannot resolve the nuances of practice.

## Appendix

### Abbreviations

EHR: Electronic health record
ER: Emergency room
GP: General practitioner
ANT: Actor–network theory
